# Supplementary material for: Rational design of a trispecific antibody targeting the HIV-1 Env with elevated anti-viral activity
Source: Nat Commun. 2018 Feb 28;9:877. doi: 10.1038/s41467-018-03335-4 (PMC5830440; doi:10.1038/s41467-018-03335-4)
Supplement: Supplementary file 1 — Supplementary Information [file 41467_2018_3335_MOESM1_ESM.pdf]

Supplementary Information

Rational Design of a Trispecific Antibody Targeting the HIV-1 Env with Elevated Anti-viral Activity

Steinhardt et al.

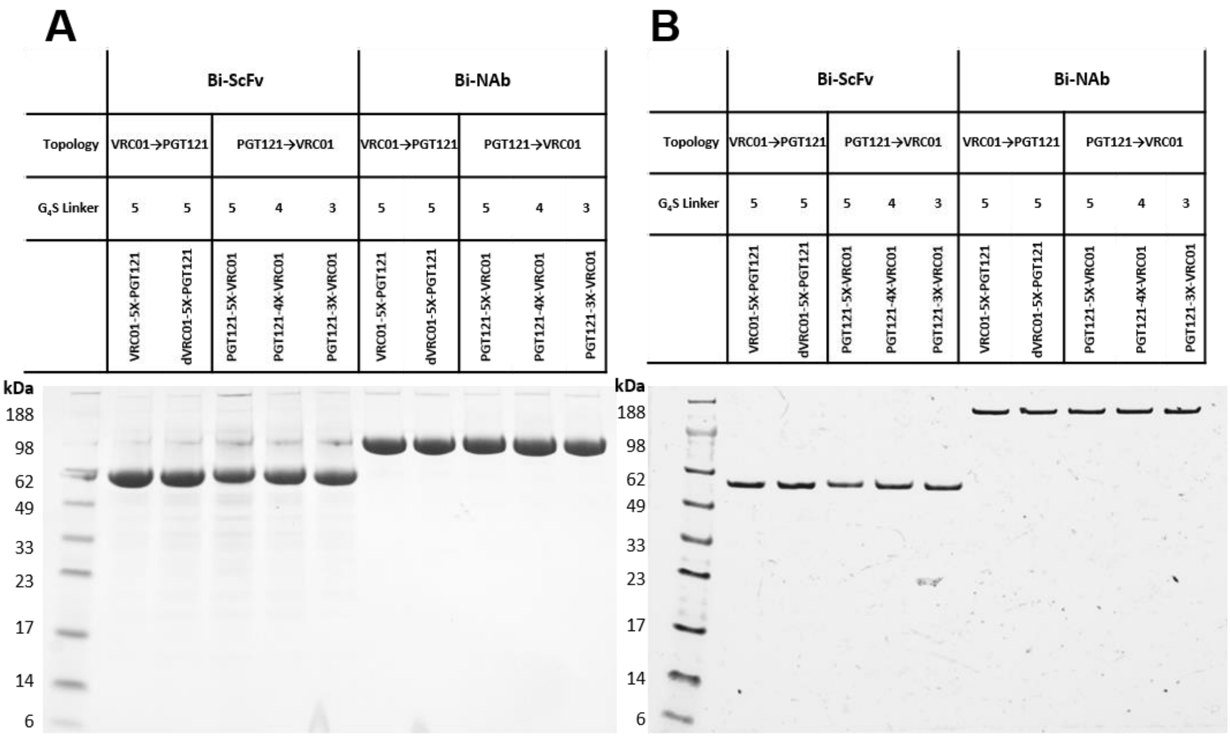

Supplementary Figure 1. Expression and purification of bispecific antibodies. (A) Reduced SDS-PAGE analysis of bispecific antibodies. (B) Non-reduced SDS page analysis of bispecific antibodies.

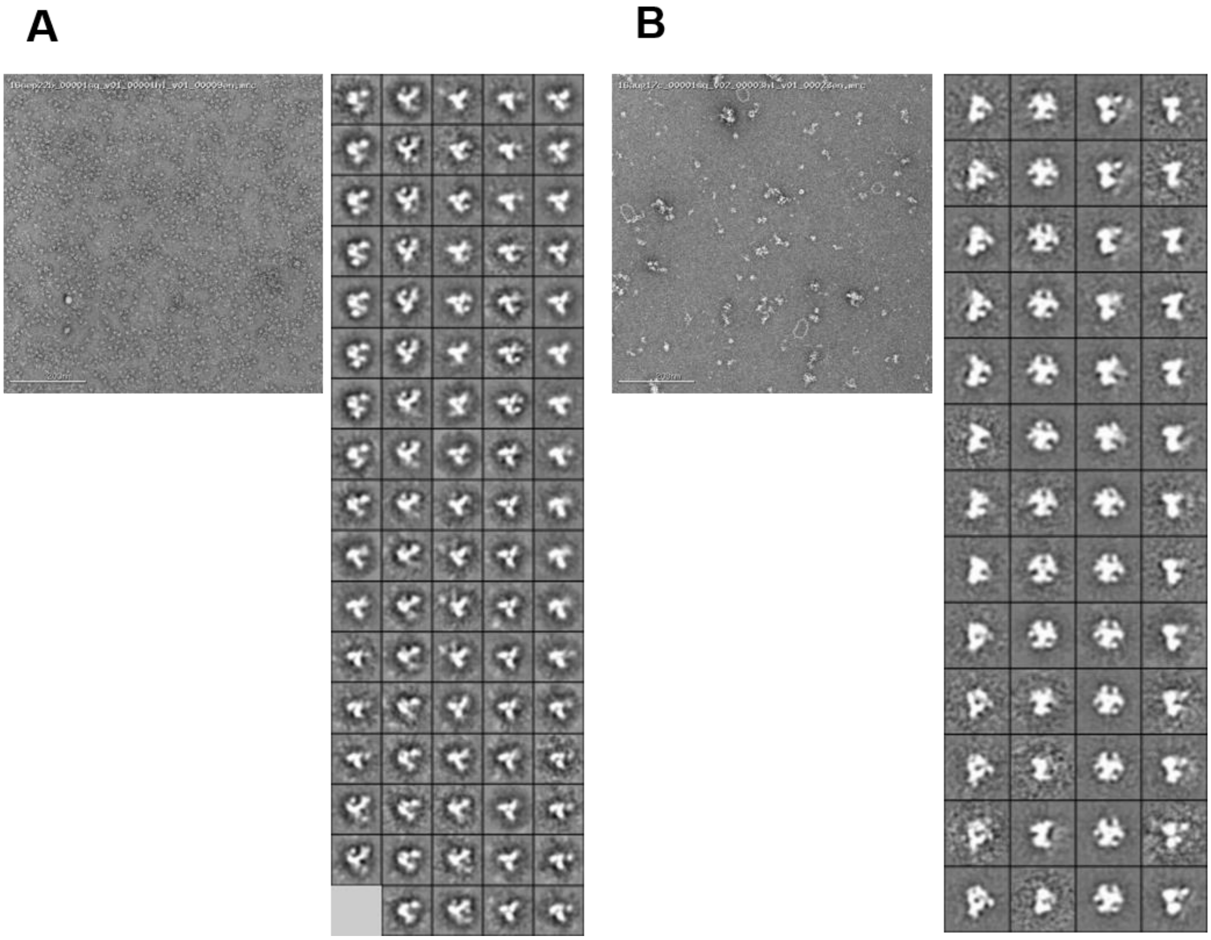

Supplementary Figure 2. **Negative stain EM of Bi-ScFv/ HIV Env trimer complex.** (A) Bi-ScFv, dVRC01-5X-PGT121, in complex with BG505.SOSIP.664 at a ratio of 0.5:1. Left, Raw micrograph; Right, 2D classes of complex. (B) Bi-ScFv, dVRC01-5X-PGT121, in complex with BG505.SOSIP.664 at a ratio of 6:1. Left, Raw micrograph; Right, 2D classes of complex.

A

Q769.d22.SG3	A	0.480	0.735	7.83	10.1	10.4	0.765	0.461	4.76	10.2	25.0	0.050	>50
6095.V1.C10.SG3	ACD	0.072	0.090	0.054	0.093	0.102	0.198	0.159	0.035	0.041	0.055	0.444	1.20
Q168.a2.SG3	AD	0.090	0.114	1.18	1.35	3.39	0.322	0.225	0.309	0.490	0.505	0.113	>50
BJOX009000.02.4.SG3	AE	0.849	1.06	0.609	1.07	1.25	2.35	1.51	0.175	0.166	0.322	3.32	7.07
242-14.SG3	AG	>50	>50	>50	>50	>50	>50	>50	>50	>50	>50	>50	>50
T251-18.SG3	AG	42.9	25.6	28.4	24.8	47.7	41.7	30.2	13.0	25.9	41.7	8.59	>50
7165.18.SG3	B	0.240	0.318	0.057	0.054	0.063	0.528	0.341	0.041	0.047	0.051	>50	0.024
AC10.29.SG3	B	0.276	0.291	0.117	0.057	0.048	0.600	0.266	0.058	0.090	0.080	2.71	0.121
BG1168.01.SG3	B	15.3	7.05	>50	>50	>50	6.08	4.61	>50	>50	>50	0.911	>50
JRFL.JB.SG3	B	0.144	0.117	0.072	0.066	0.081	0.174	0.175	0.090	0.119	0.102	0.022	0.029
QH0692.42.SG3	B	6.09	5.67	1.22	1.12	1.44	8.02	6.67	0.872	1.08	1.61	3.82	7.01
3637.V5.C3.SG3	C	>50	>50	>50	>50	>50	>50	>50	>50	>50	>50	14.9	>50
CAP210.E8.SG3	C	18.3	9.15	39.9	35.4	>50	13.2	11.4	4.69	2.40	4.11	>50	48.2
DU172.17.SG3	C	1.06	0.930	0.201	0.213	0.243	0.965	0.883	0.178	0.207	0.254	>50	0.089
DU422.01.SG3	C	0.276	0.273	0.135	0.168	0.177	0.351	0.378	0.068	0.074	0.091	>50	0.195
TZA125.17.SG3	C	9.21	10.3	15.8	9.96	16.5	8.69	12.1	1.61	1.72	2.64	>50	14.2
ZM214.15.SG3	C	1.33	2.13	0.885	0.822	0.423	5.31	2.46	0.536	1.07	0.741	0.590	0.661
ZM249.1.SG3	C	0.654	0.675	10.4	13.5	18.9	0.923	0.844	8.55	11.1	>50	0.100	>50
57128.vrc15.SG3	D	3.06	1.69	>50	31.5	45.6	2.76	2.26	0.838	0.789	1.36	>50	4.74
X2088.c9.SG3	G	0.102	0.087	0.015	0.036	0.036	0.186	0.077	0.016	0.015	0.031	>50	0.002

B

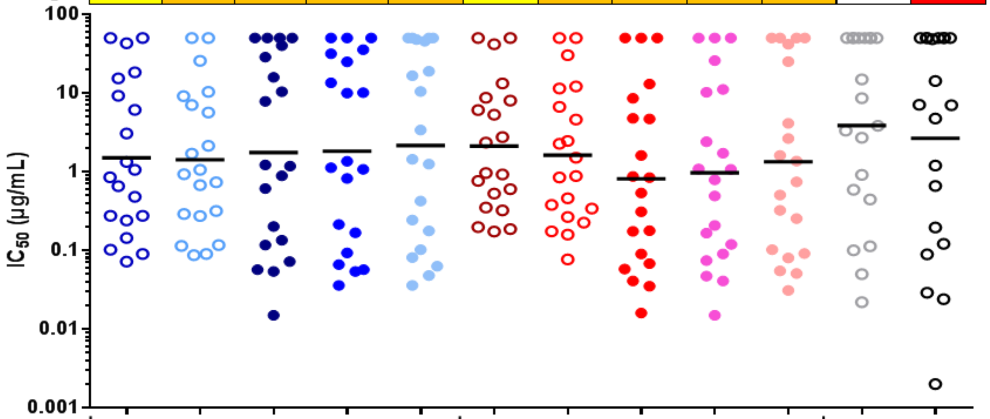

	Bi-ScFv†					Bi-NAb					Parental Ab	
Topology	VRC01→PGT121		PGT121→VRC01			VRC01→PGT121		PGT121→VRC01			NA	NA
G_4S Linker	5	5	5	4	3	5	5	5	4	3	NA	NA
	VRC01-5X-PGT121	dVRC01-5X-PGT121	PGT121-5X-VRC01	PGT121-4X-VRC01	PGT121-3X-VRC01	VRC01-5X-PGT121	dVRC01-5X-PGT121	PGT121-5X-VRC01	PGT121-4X-VRC01	PGT121-3X-VRC01	VRC01	PGT121
# Viruses	20	20	20	20	20	20	20	20	20	20	20	20
Total Virus Neutralized	18	18	16	17	16	18	18	17	17	16	12	13
Breadth (%)	90	90	80	85	80	90	90	85	85	80	60	65
IC_{50} GMT (µg/mL)	1.017	0.956	0.758	1.012	0.978	1.486	1.106	0.392	0.483	0.538	0.697	0.545
Total IC_{50} GMT (µg/mL)	1.501	1.420	1.752	1.817	2.149	2.113	1.619	0.812	0.969	1.331	3.849	2.650

C

		dVRC01-5X-PGT121	Parental Ab		
Virus ID	clade	Bi-NAb	VRC01	PGT121	VRC01+ PGT121
T250-4	AG	0.051	>25	0.013	0.091
BJOX002000.03.2	BC	0.275	>25	0.133	0.684
DU172.17	C	0.406	>25	0.422	1.83
DU422.01	C	0.426	>25	0.136	0.839
TV1.29	C	0.814	>25	0.266	1.76
TZA125.17	C	4.84	>25	15.6	>50
57128.vrc15	D	0.985	>25	2.99	12.4
X2088.c9	G	0.081	>25	0.018	0.131
6631.V3.C10	C	18.0	>25	>25	>50
CAP210.E8	C	14.7	>25	>25	>50
3817.v2.c59	CD	48.5	>25	>25	>50

D

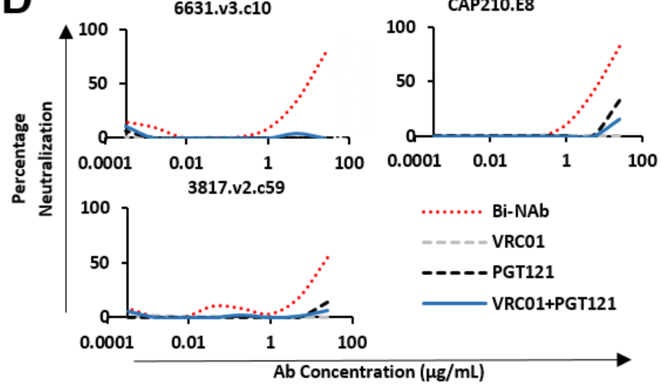

Supplementary Figure 3. Neutralization profile of bispecific antibodies tested with a 20-virus panel. (A) Summary of IC_{50} (μg/ml) for bispecific antibodies tested in 20 virus panel. (B) Scatter plots of IC_{50} titers in which each virus is represented by an individual circle. († indicates that the IC_{50} was adjusted by a factor of 3 to account for the molarity difference between the lower molecular weight Bi-ScFv and the IgG and Bi-NAb). (C) Summary of IC_{50} titers (μg/ml) against VRC01- and dual-resistant viruses that are sensitive to the Bi-NAb in a confirmatory experiment, with individual parental bNAb and a cocktail of parental bNAb (VRC01+PGT121) as control. (D) Raw neutralization curves of dual resistant viruses sensitive to the Bi-NAb in (C).

A

B

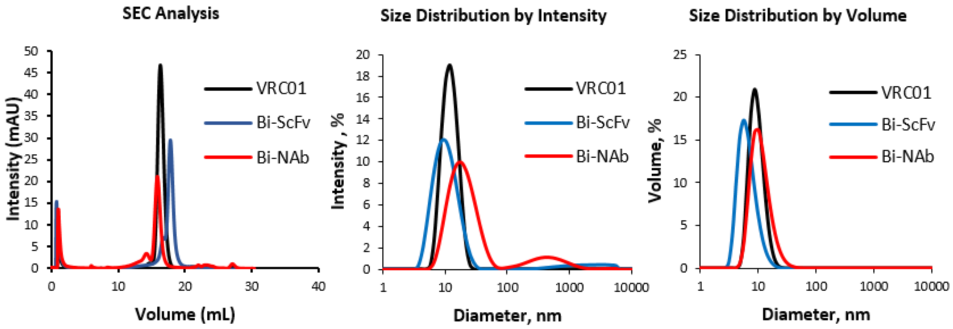

C

	Z-Average	PdI
	d.nm	
VRC01	11.19	0.062
Bi-ScFv	9.75	0.236
Bi-NAb	19.19	0.321

D

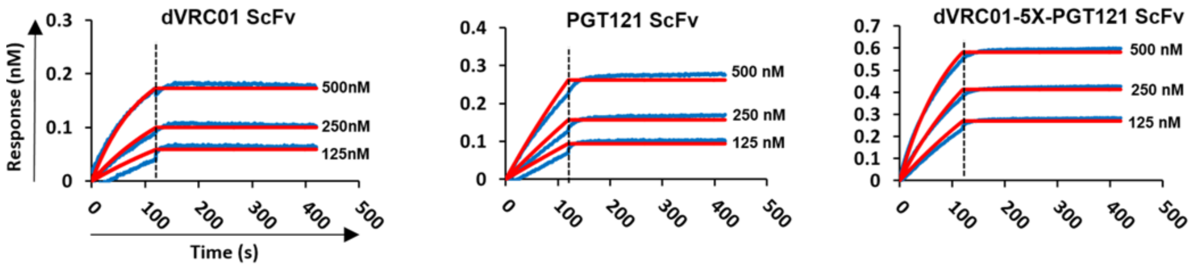

E

	K_D (M)	k_{on}(1/Ms)	K_{off}(1/s)
dVRC01 ScFv	<1.0E-12	2.22E+04	<1.0E-07
PGT121 ScFv	<1.0E-12	4.92E+03	<1.0E-07
dVRC01-5X-PGT121 ScFv	<1.0E-12	2.08E+04	<1.0E-07

Supplementary Figure 4. Biochemical properties of dVRC01-5X-PGT121 Bi-ScFv and Bi-NAb. (A) Analytical size exclusion chromatography profiles of Bi-ScFv and Bi-NAb, with VRC01 as a control. (B) Dynamic light scattering (DLS) profiles of Bi-ScFv and Bi-NAb. (C) DLS parameters of Bi-ScFv and Bi-NAb, with VRC01 as control. PdI, polydispersity index. (D) BLI curves of ScFv binding to biotinylated BG505 SOSIP.664. Streptavidin biosensors were loaded with biotinylated BG505 SOSIP.664 followed by determination of association and dissociation rates for respective antibodies using concentrations of scFvs at 500 nM (top curve), 250 nM (middle curve) and 125 nM (lower curve). (E) K_D, k_{on}, k_{off} values for dVRC01 ScFv, PGT121 ScFv and dVRC01-5X-PGT121 ScFv binding to BG505 SOSIP.664 trimer.

Supplementary Figure 5

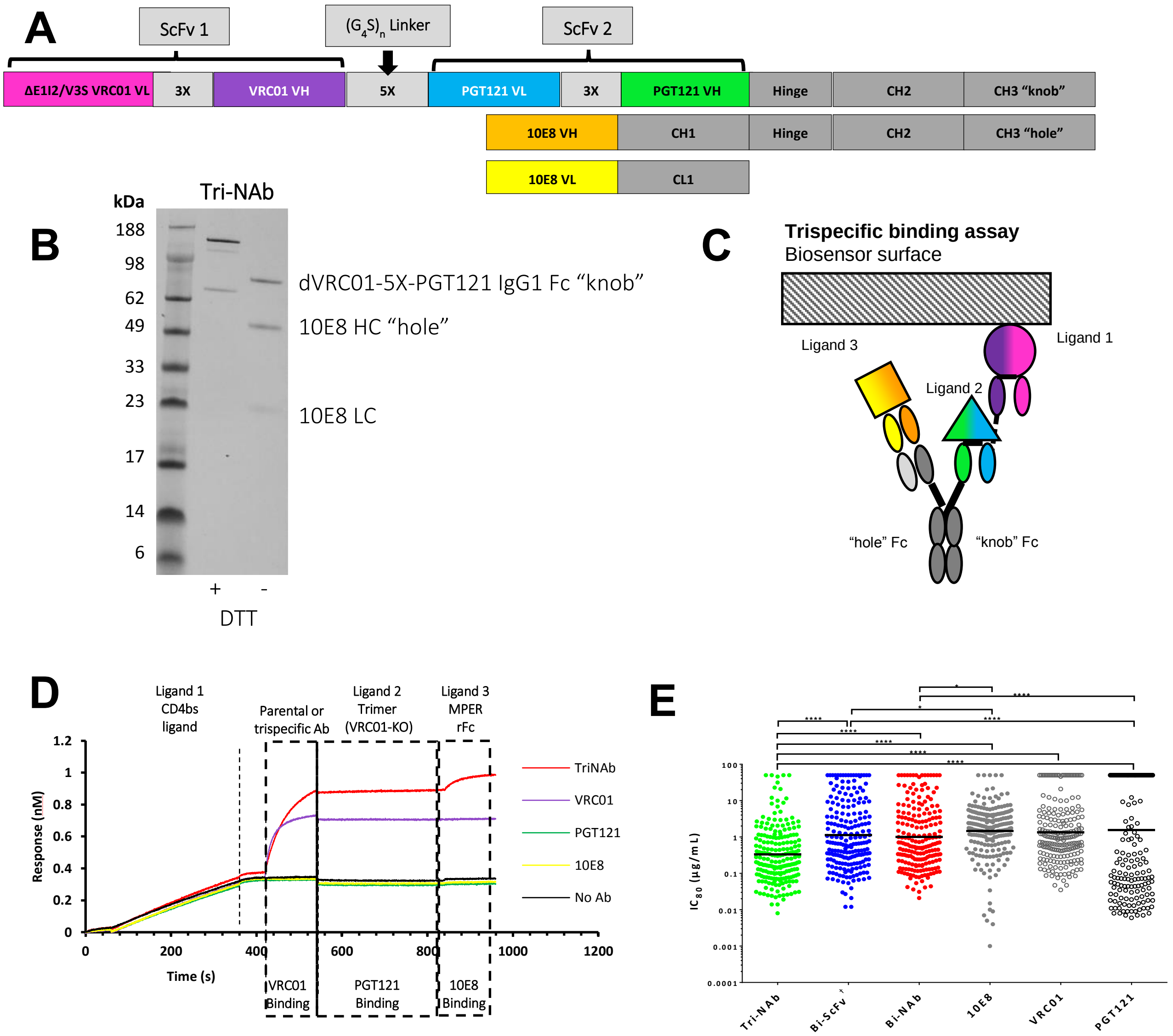

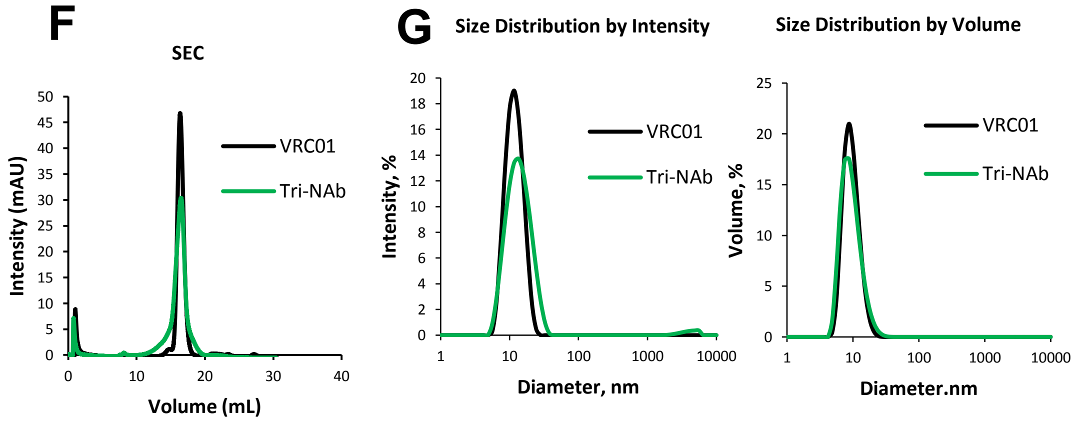

H

	Z-Average d.nm	PdI
VRC01	11.19	0.062
Tri-NAb	12.82	0.173

Supplementary Figure 5. Tri-NAb construct, expression, and characterization. (A) Tri-NAb constructs scheme. (B) Reduced (left lane) and non-reduced (right lane) SDS-PAGE analysis of trispecific antibody. (C) Scheme of the trispecific binding assay via biolayer interferometry (BLI). (D) BLI curves of trispecific binding assay. OCTET biosensors were loaded with ligand 1 (biotinylated RSC3) specific for VRC01 epitope (CD4bs), followed by trispecific antibody, ligand 2 (BG505 SOSIP.664_D368R) specific for PGT121 epitope (V3 glycan), and ligand 3 (MPER rFc) specific for 10E8 epitope (MPER). Parental IgGs were used as control. (E) Scatter plots of IC_{80} titers in which each virus is represented by an individual circle (Statistical differences in neutralization were evaluated using non-parametric t test (Wilcoxon matched-pairs signed rank test) with *p<0.05, **p<0.01, ***p<0.001, ****p<0.0001). († indicates that the IC_{80} titer was adjusted by a factor of 3 to account for the molarity difference between the lower molecular weight Bi-ScFv and the IgG, Bi-NAb and Tri-NAb). (F) Analytical size exclusion chromatography profile of Tri-NAb with VRC01 as control. (G) Dynamic light scattering (DLS) profiles, and (H) DLS parameters of Tri-NAb with VRC01 as control.

Supplementary Figure 6

Virus ID	Clade	Tri-NAb	Bi-ScFv	Bi-NAb	10E8	VRC01	PGT121
0260.v5.c36	A	0.170	0.124	0.416	9.87	0.523	0.039
0330.v4.c3	A	0.092	0.082	0.241	1.12	0.066	0.041
0439.v5.c1	A	0.471	1.57	2.93	1.23	0.228	>50
3365.v2.c20	A	0.078	0.070	0.232	1.60	0.060	0.059
3415.v1.c1	A	0.312	0.354	0.847	4.69	0.087	>50
3718.v3.c11	A	0.147	0.116	0.295	0.838	0.411	1.40
398-F1_F6_20	A	0.005	0.015	0.147	0.704	0.102	0.002
BB201.B42	A	0.049	0.015	0.063	0.613	0.262	0.003
BB539.2B13	A	0.296	1.82	1.29	0.591	0.105	>50
BG505.W6M.C2	A	0.089	0.048	0.134	0.689	0.037	0.032
BI369.9A	A	0.072	0.087	0.103	0.356	0.047	0.008
BS208.B1	A	0.026	0.061	0.096	0.319	0.027	>50
KER2008.12	A	0.131	0.067	0.233	>50	0.487	2.22
KER2018.11	A	0.211	0.064	0.271	1.89	0.348	>50
KNH1209.18	A	0.034	0.014	0.049	0.406	0.119	0.002
MB201.A1	A	0.030	0.008	0.035	0.411	0.241	0.005
MB539.2B7	A	0.772	1.94	4.16	>50	0.512	>50
MI369.A5	A	0.107	0.056	0.155	0.671	0.236	0.022
MS208.A1	A	0.405	0.504	0.994	0.187	0.174	>50
Q23.17	A	0.038	0.060	0.131	0.461	0.099	0.004
Q259.17	A	0.042	0.013	0.043	4.76	0.085	>50
Q769.d22	A	0.315	0.175	0.489	1.91	0.036	>50
Q769.h5	A	0.229	0.094	0.325	2.89	0.072	>50
Q842.d12	A	0.048	0.030	0.139	2.82	0.034	0.016
QH209.14M.A2	A	0.168	0.175	0.456	1.30	0.026	>50
RW020.2	A	0.021	0.006	0.032	0.902	0.217	0.002
UG037.8	A	0.092	0.104	0.333	0.048	0.073	0.065
246-F3.C10.2	AC	0.037	0.028	0.092	0.210	0.254	>50
3301.V1.C24	AC	0.070	0.029	0.085	2.97	0.095	0.009
3589.V1.C4	AC	0.208	0.127	0.475	5.77	0.081	>50
6540.v4.c1	AC	28.7	>50	>50	2.24	>50	>50
6545.V4.C1	AC	5.56	>50	>50	2.54	>50	>50
0815.V3.C3	ACD	0.058	0.031	0.109	0.491	0.029	0.020
6095.V1.C10	ACD	0.012	0.146	0.331	0.0005	0.631	37.3
3468.V1.C12	AD	0.025	0.015	0.045	0.381	0.058	0.042
Q168.a2	AD	0.136	0.067	0.141	0.463	0.101	>50
Q461.e2	AD	4.41	3.28	6.73	2.29	0.420	>50
620345.c1	AE	26.3	>50	>50	0.989	>50	>50
BJOX009000.02.4	AE	0.043	0.228	0.654	0.251	1.74	14.7
BJOX010000.06.2	AE	0.177	4.85	18.8	0.060	8.40	>50
BJOX025000.01.1	AE	0.357	>50	>50	0.228	20.2	>50
BJOX028000.10.3	AE	0.076	0.152	0.285	0.167	0.188	>50
C1080.c3	AE	0.077	6.87	5.02	0.108	2.63	>50
C2101.c1	AE	0.138	1.47	1.86	1.20	0.269	>50
C3347.c11	AE	0.005	1.77	1.73	0.019	0.213	>50
C4118.09	AE	0.113	2.00	2.26	0.421	0.285	>50
CM244.ec1	AE	0.117	0.237	0.867	0.365	0.116	>50
CNE3	AE	3.85	>50	>50	1.37	1.79	>50
CNE5	AE	0.183	1.01	4.59	1.17	0.398	>50
CNE55	AE	0.233	1.78	5.74	0.038	0.358	>50
CNE56	AE	0.476	2.74	6.54	0.060	0.525	>50
CNE59	AE	0.065	2.83	2.26	0.001	0.368	>50
CNE8	AE	0.005	0.356	1.14	0.140	0.299	>50
M02138	AE	0.031	4.92	7.18	0.014	0.898	>50
R1166.c1	AE	0.035	4.58	10.6	0.488	2.09	>50
R2184.c4	AE	0.557	0.791	2.33	0.576	0.106	>50
R3265.c6	AE	0.894	2.87	6.81	1.58	0.382	>50
TH023.6	AE	0.015	5.07	1.05	0.0003	0.546	>50
TH966.8	AE	0.027	2.29	3.47	0.039	0.390	>50
TH976.17	AE	0.256	1.05	2.96	0.392	0.299	>50
235-47	AG	0.067	0.071	0.246	0.244	0.043	0.110
242-14	AG	1.23	33.2	>50	0.568	>50	>50
263-8	AG	0.227	0.250	0.683	0.229	0.176	1.23
269-12	AG	0.083	0.056	0.239	0.124	0.313	0.164
271-11	AG	0.020	0.008	0.032	0.891	0.059	11.7
928-28	AG	0.088	1.16	2.63	0.079	0.394	31.0
DJ263.8	AG	0.006	0.016	0.100	0.009	0.047	0.064
T250-4	AG	0.025	0.007	0.028	1.07	>50	0.001
T251-18	AG	0.052	2.86	8.32	0.666	4.21	10.8

IC_{50} (µg/mL)
<0.001
.001-.01
.01-.100
.100-1.00
1.00-10.0
>10.0

Supplementary Figure 6

Virus ID	Clade	Tri-NAb	Bi-ScFv	Bi-NAb	10E8	VRC01	PGT121
T253-11	AG	0.239	0.217	2.29	1.21	0.397	>50
T255-34	AG	0.013	0.034	0.089	0.228	0.500	>50
T257-31	AG	0.247	0.480	1.71	0.336	1.72	>50
T266-60	AG	0.684	0.534	1.40	>50	1.81	0.160
T278-50	AG	2.36	>50	>50	0.357	>50	>50
T280-5	AG	0.024	0.014	0.050	0.715	0.032	0.002
T33-7	AG	0.105	0.088	0.180	0.818	0.018	>50
3988.25	B	0.033	0.021	0.058	0.070	0.494	0.002
5768.04	B	0.124	0.042	0.108	1.63	0.365	0.039
6101.10	B	0.010	0.018	0.036	0.0010	0.035	0.002
6535.3	B	0.007	0.006	0.031	0.190	1.93	0.003
7165.18	B	0.055	0.085	0.196	0.659	28.2	0.019
45_01dG5	B	0.015	0.008	0.051	0.106	0.018	0.002
89.6.DG	B	0.007	0.124	0.160	0.318	0.762	0.016
AC10.29	B	0.128	0.179	0.534	0.102	1.81	0.028
ADA.DG	B	0.022	0.075	0.193	0.055	0.470	0.002
Bal.01	B	0.031	0.007	0.028	0.421	0.095	0.011
BaL.26	B	0.036	0.023	0.060	0.518	0.042	0.010
BG1168.01	B	0.580	2.03	2.27	0.396	0.869	>50
BL01.DG	B	4.01	>50	>50	0.362	>50	>50
BR07.DG	B	0.031	0.162	0.338	0.118	1.57	0.064
BX08.16	B	0.020	0.059	0.052	0.213	0.274	0.002
CAAN.A2	B	0.058	0.052	0.104	1.45	1.03	0.005
CNE10	B	0.018	0.024	0.090	0.014	0.565	0.005
CNE12	B	0.021	0.023	0.073	0.301	0.866	0.002
CNE14	B	0.018	0.012	0.048	0.151	0.275	0.002
CNE4	B	0.063	0.353	0.966	0.059	0.910	11.5
CNE57	B	0.029	0.050	0.155	0.059	0.563	0.008
HO86.8	B	5.94	>50	>50	0.326	>50	>50
HT593.1	B	0.094	0.445	1.10	0.049	0.476	>50
HXB2.DG	B	0.008	0.003	0.065	0.003	0.034	>50
JRCSF.JB	B	0.158	0.036	0.278	0.429	0.362	0.061
JRFL.JB	B	0.053	0.021	0.087	0.174	0.028	0.017
MN.3	B	0.006	0.009	0.057	0.0003	0.020	>50
PVO.04	B	0.732	0.497	1.28	1.60	0.511	0.132
QH0515.01	B	0.535	0.462	1.29	2.25	1.01	8.70
QH0692.42	B	0.331	0.966	1.70	0.531	1.54	0.940
REJO.67	B	0.072	0.046	0.178	0.302	0.075	8.87
RHPA.7	B	0.065	0.035	0.110	1.01	0.034	0.014
SC422.8	B	0.166	0.167	0.488	0.343	0.127	0.098
SF162.LS	B	0.010	***0.0006***	0.017	0.245	0.207	0.004
SS1196.01	B	0.002	0.017	0.063	0.244	0.304	0.002
THRO.18	B	0.316	1.70	2.60	0.092	3.16	>50
TRJO.58	B	0.377	0.321	0.820	1.13	0.101	4.31
TRO.11	B	0.033	0.033	0.138	0.028	0.469	0.006
WITO.33	B	0.086	0.298	0.893	0.031	0.102	0.787
X2278.C2.B6	B	0.051	0.064	0.188	0.442	0.151	0.007
YU2.DG	B	0.049	0.047	0.200	1.17	0.076	0.068
BJOX002000.03.2	BC	0.066	0.048	0.132	0.384	>50	0.018
CH038.12	BC	0.024	0.043	0.104	0.271	0.447	0.004
CH070.1	BC	0.002	0.039	0.096	6.65	14.0	0.003
CH117.4	BC	0.018	0.008	0.059	0.270	0.105	>50
CH119.10	BC	0.023	0.070	0.173	0.591	0.833	0.029
CH181.12	BC	0.063	0.048	0.124	0.754	0.487	0.007
CNE15	BC	0.009	0.105	0.359	0.844	0.141	19.0
CNE19	BC	0.007	0.002	0.020	0.251	0.247	0.007
CNE20	BC	0.018	0.002	0.017	0.131	7.39	0.002
CNE21	BC	0.031	0.026	0.098	0.979	0.274	0.004
CNE40	BC	0.005	0.234	0.440	0.0010	0.433	0.224
CNE7	BC	0.033	0.055	0.175	0.130	0.187	0.032
286.36	C	0.009	0.005	0.037	1.19	0.223	0.002
288.38	C	0.007	0.030	0.085	0.435	1.38	0.006
0013095-2.11	C	0.004	0.306	0.864	0.009	0.086	>50
001428-2.42	C	0.038	0.018	0.085	1.71	0.014	0.023
0077_V1.C16	C	0.171	0.224	0.482	1.86	1.13	>50
00836-2.5	C	0.024	0.005	0.047	0.666	0.122	31.8
0921.V2.C14	C	0.093	0.050	0.135	0.908	0.230	>50
16055-2.3	C	0.047	0.016	0.070	1.10	0.100	1.02
16845-2.22	C	0.068	2.99	5.21	0.020	2.95	9.41

IC_{50} (µg/mL)
<0.001
.001-.01
.01-.100
.100-1.00
1.00-10.0
>10.0

Supplementary Figure 6

Virus ID	Clade	Tri-NAb	Bi-ScFv	Bi-NAb	10E8	VRC01	PGT121
16936-2.21	C	0.003	0.011	0.038	0.264	0.154	0.003
25710-2.43	C	0.037	0.034	0.154	0.064	0.487	0.014
25711-2.4	C	0.063	0.028	0.103	0.516	0.559	0.010
25925-2.22	C	0.072	0.060	0.188	0.402	0.550	0.024
26191-2.48	C	0.136	0.107	0.406	1.83	0.183	0.150
3168.V4.C10	C	0.544	0.638	1.68	2.83	0.129	0.485
3637.V5.C3	C	2.07	11.4	18.9	2.12	1.97	>50
3873.V1.C24	C	0.007	0.322	1.48	5.51	2.81	0.015
426c	C	0.429	0.419	0.953	0.445	1.93	>50
6322.V4.C1	C	1.67	15.2	5.00	0.923	>50	>50
6471.V1.C16	C	>50	>50	>50	4.98	>50	>50
6631.V3.C10	C	1.39	1.91	2.63	0.934	>50	>50
6644.V2.C33	C	0.027	0.021	0.143	0.013	0.153	0.018
6785.V5.C14	C	0.134	0.059	0.149	0.701	0.253	0.019
6838.V1.C35	C	0.012	0.002	0.015	0.292	0.288	0.119
96ZM651.02	C	0.011	0.026	0.070	0.033	0.807	0.009
BR025.9	C	0.014	0.019	0.033	0.307	0.528	0.002
CAP210.E8	C	0.526	2.55	4.88	0.474	>50	>50
CAP244.D3	C	0.015	0.455	1.28	0.369	1.34	>50
CAP256.206.C9	C	0.049	0.054	0.102	0.713	1.07	0.010
CAP45.G3	C	0.168	0.166	0.373	0.722	6.75	2.08
Ce1176.A3	C	0.071	0.051	0.120	0.252	1.85	0.016
CE703010217.B6	C	0.018	0.009	0.038	0.096	0.195	0.002
CNE30	C	0.094	0.224	0.609	0.456	0.693	0.061
CNE31	C	1.01	0.713	2.30	1.32	0.772	0.789
CNE53	C	0.010	0.050	0.106	0.213	0.112	0.022
CNE58	C	0.366	0.970	2.32	0.229	0.252	>50
DU123.06	C	0.002	0.053	0.199	0.132	5.70	0.033
DU151.02	C	0.059	0.011	0.044	0.461	10.5	0.005
DU156.12	C	0.015	0.005	0.036	0.023	0.077	0.005
DU172.17	C	0.039	0.059	0.140	0.057	>50	0.104
DU422.01	C	0.182	0.057	0.187	0.224	>50	0.164
MW965.26	C	0.005	0.012	0.051	0.0010	0.043	0.011
SO18.18	C	0.0010	0.004	0.023	1.60	0.052	0.002
TV1.29	C	1.17	0.319	0.473	0.248	>50	0.118
TZA125.17	C	0.428	2.11	5.14	0.217	>50	9.96
TZBD.02	C	0.017	0.007	0.035	1.41	0.043	0.005
ZA012.29	C	0.060	0.023	0.062	1.47	0.327	0.005
ZM106.9	C	0.049	0.023	0.089	>50	0.264	0.005
ZM109.4	C	0.060	0.091	0.351	0.161	0.142	13.7
ZM135.10a	C	0.004	1.20	2.12	0.033	1.40	1.50
ZM176.66	C	0.030	0.071	0.221	0.267	0.045	13.8
ZM197.7	C	0.161	1.14	4.15	0.055	0.532	>50
ZM214.15	C	0.533	0.857	2.30	2.22	0.957	0.682
ZM215.8	C	0.023	0.021	0.100	0.044	0.362	0.014
ZM233.6	C	0.090	0.070	0.268	0.270	1.98	4.14
ZM249.1	C	0.279	0.486	0.810	0.830	0.107	>50
ZM53.12	C	0.011	0.0008	0.010	2.62	0.702	0.002
ZM55.28a	C	0.212	0.137	0.407	2.34	0.241	0.070
3326.V4.C3	CD	0.018	0.008	0.017	1.40	0.107	>50
3337.V2.C6	CD	0.039	0.051	0.110	1.09	0.105	21.1
3817.v2.c59	CD	0.230	7.23	11.6	0.229	>50	>50
191821.E6.1	D	0.921	0.487	1.57	1.91	0.438	>50
231965.c1	D	0.261	1.31	1.41	11.0	0.392	>50
247-23	D	0.639	19.5	18.3	0.344	1.63	>50
3016.v5.c45	D	0.037	0.043	0.078	0.710	0.117	>50
57128.vrc15	D	0.098	0.438	0.875	0.212	>50	2.16
6405.v4.c34	D	0.103	0.092	0.259	0.461	1.69	0.019
A03349M1.vrc4a	D	0.189	0.071	0.194	0.270	4.42	0.013
A07412M1.vrc12	D	0.041	0.011	0.057	0.140	0.101	0.012
NKU3006.ec1	D	1.91	3.28	8.54	0.673	0.460	>50
UG021.16	D	0.016	0.275	0.340	0.046	0.451	2.41
UG024.2	D	0.035	2.28	0.830	0.053	0.219	>50
P0402.c2.11	G	0.0010	0.024	0.081	0.057	0.207	0.004
P1981.C5.3	G	0.025	0.006	0.024	0.024	0.336	0.004
X1193.c1	G	0.009	0.128	0.239	0.341	0.124	0.028
X1254.c3	G	0.010	0.072	0.188	3.67	0.055	0.024
X1632.S2.B10	G	0.065	0.088	0.178	0.387	0.131	>50
X2088.c9	G	0.017	0.016	0.029	>50	>50	0.003

IC_{50} (µg/mL)
<0.001
.001-.01
.01-.100
.100-1.00
1.00-10.0
>10.0

Supplementary Figure 6

Virus ID	Clade	Tri-NAb	Bi-ScFv	Bi-NAb	10E8	VRC01	PGT121
X2131.C1.B5	G	0.016	0.023	0.079	0.039	0.467	0.010
SIVmac251.30.SG3	NA	>50	>50	>50	>50	>50	>50
SVA.MLV	NA	>50	>50	>50	>50	>50	>50

	Tri-NAb	Bi-ScFv	Bi-NAb	10E8	VRC01	PGT121
**# Viruses**	208	208	208	208	208	208
**Total Virus Neutralized**						
**IC50 <50 (µg/mL)**	207	199	198	203	188	133
**IC50 <10 (µg/mL)**	205	195	193	202	184	122
**IC50 <1.0 (µg/mL)**	192	162	147	152	152	108
**IC50 <0.1 (µg/mL)**	131	112	58	42	40	93
**IC50 <0.01 (µg/mL)**	24	23	0	10	0	47
**% Virus Neutralized**						
**IC50 <50 (µg/mL)**	99.5	95.7	95.2	97.6	90.4	63.9
**IC50 <10 (µg/mL)**	98.6	93.8	92.8	97.1	88.5	58.7
**IC50 <1.0 (µg/mL)**	92.3	77.9	70.7	73.1	73.1	51.9
**IC50 <0.1 (µg/mL)**	63.0	53.8	27.9	20.2	19.2	44.7
**IC50 <0.01(µg/mL)**	11.5	11.1	0.0	4.8	0.0	22.6
**Median IC50 (µg/mL)**	0.063	0.071	0.198	0.039	0.287	0.019
**Geometric Mean (µg/mL)**	0.069	0.108	0.297	0.039	0.301	0.045

IC_{50} (µg/mL)
<0.001
.001-.01
.01-.100
.100-1.00
1.00-10.0
>10.0

Supplementary Figure 6. Summary of IC_{50} (µg/ml) for trispecific and bispecific antibodies tested in 208 virus panel.

Supplementary Figure 7

		Tri-NAb	Bi-ScFv	Bi-NAb	10E8	VRC01	PGT121
0260.v5.c36	A	0.716	0.378	0.958	21.7	1.35	0.143
0330.v4.c3	A	0.403	0.216	0.619	3.64	0.202	0.194
0439.v5.c1	A	2.27	2.60	7.07	3.95	0.438	>50
3365.v2.c20	A	0.301	0.150	0.534	4.56	0.125	1.34
3415.v1.c1	A	1.40	0.752	2.18	11.5	0.177	>50
3718.v3.c11	A	0.573	0.337	0.685	4.42	5.58	8.64
398-F1_F6_20	A	0.027	0.049	0.322	6.17	0.479	0.011
BB201.B42	A	0.161	0.053	0.137	1.96	0.614	0.011
BB539.2B13	A	1.97	3.17	3.46	13.0	0.407	>50
BG505.W6M.C2	A	0.293	0.130	0.338	2.14	0.125	0.256
BI369.9A	A	0.340	0.418	0.563	1.29	0.532	0.043
BS208.B1	A	0.114	0.139	0.245	3.27	0.080	>50
KER2008.12	A	0.421	0.221	0.673	>50	1.46	>50
KER2018.11	A	0.979	0.213	0.800	7.16	0.976	>50
KNH1209.18	A	0.101	0.037	0.110	2.39	0.298	0.007
MB201.A1	A	0.077	0.025	0.089	1.36	0.452	0.026
MB539.2B7	A	2.45	4.72	11.0	>50	1.24	>50
MI369.A5	A	0.642	0.204	0.509	1.77	0.843	0.087
MS208.A1	A	1.79	1.94	3.39	1.14	0.593	>50
Q23.17	A	0.149	0.117	0.352	1.60	0.212	0.019
Q259.17	A	0.147	0.032	0.080	12.0	0.242	>50
Q769.d22	A	1.11	0.435	1.14	4.47	0.098	>50
Q769.h5	A	0.650	0.223	0.703	7.44	0.145	>50
Q842.d12	A	0.169	0.096	0.351	7.58	0.075	0.047
QH209.14M.A2	A	0.705	0.577	1.48	4.09	0.094	>50
RW020.2	A	0.073	0.021	0.076	2.92	0.647	0.009
UG037.8	A	0.483	0.339	0.990	0.353	0.186	0.237
246-F3.C10.2	AC	0.181	0.108	0.301	1.49	0.650	>50
3301.V1.C24	AC	0.207	0.070	0.170	9.50	0.223	0.030
3589.V1.C4	AC	0.709	0.315	1.10	11.7	0.199	>50
6540.v4.c1	AC	>50	>50	>50	7.01	>50	>50
6545.V4.C1	AC	31.4	>50	>50	7.50	>50	>50
0815.V3.C3	ACD	0.186	0.081	0.356	1.81	0.085	0.072
6095.V1.C10	ACD	0.048	0.557	0.938	0.004	2.04	>50
3468.V1.C12	AD	0.072	0.040	0.101	2.04	0.117	1.05
Q168.a2	AD	0.382	0.127	0.377	2.88	0.230	>50
Q461.e2	AD	11.5	7.51	17.0	4.68	1.02	>50
620345.c1	AE	>50	>50	>50	3.73	>50	>50
BJOX009000.02.4	AE	0.279	0.762	1.92	1.47	4.80	>50
BJOX010000.06.2	AE	1.70	15.5	>50	0.476	20.9	>50
BJOX025000.01.1	AE	2.98	>50	>50	1.54	>50	>50
BJOX028000.10.3	AE	0.432	0.804	1.01	0.876	1.03	>50
C1080.c3	AE	0.973	18.1	22.4	0.613	9.98	>50
C2101.c1	AE	0.816	3.46	4.64	4.12	0.581	>50
C3347.c11	AE	0.060	3.82	3.88	0.089	0.452	>50
C4118.09	AE	1.04	5.11	7.21	2.30	0.720	>50
CM244.ec1	AE	0.913	0.741	2.48	1.46	0.452	>50
CNE3	AE	20.9	>50	>50	4.01	11.0	>50
CNE5	AE	1.67	3.00	11.0	2.52	0.914	>50
CNE55	AE	1.44	4.29	17.1	0.605	0.933	>50
CNE56	AE	2.06	7.51	28.5	0.314	1.30	>50
CNE59	AE	0.339	11.8	9.43	0.010	1.70	>50
CNE8	AE	0.025	1.46	4.45	1.42	0.965	>50
M02138	AE	0.135	21.4	25.8	0.126	3.02	>50
R1166.c1	AE	0.463	13.7	44.6	2.02	5.39	>50
R2184.c4	AE	1.94	2.26	6.10	2.20	0.288	>50
R3265.c6	AE	4.97	9.74	19.7	9.28	1.41	>50
TH023.6	AE	0.115	21.1	4.46	0.034	5.75	>50
TH966.8	AE	0.180	5.60	12.7	0.291	0.983	>50
TH976.17	AE	1.18	2.93	7.63	1.75	0.726	>50
235-47	AG	0.309	0.265	0.857	0.786	0.154	0.842
242-14	AG	8.76	>50	>50	3.17	>50	>50
263-8	AG	1.00	0.674	2.34	0.991	0.536	7.73
269-12	AG	0.308	0.155	0.632	0.475	0.679	1.26
271-11	AG	0.078	0.028	0.096	4.34	0.200	>50
928-28	AG	0.677	2.92	7.95	0.365	0.968	>50
DJ263.8	AG	0.061	0.090	0.328	0.100	0.490	0.202
T250-4	AG	0.092	0.024	0.078	3.45	>50	0.012
T251-18	AG	0.951	8.52	26.9	2.55	12.0	>50
T253-11	AG	1.16	0.603	5.03	4.05	1.23	>50
T255-34	AG	0.160	0.141	0.312	1.14	1.31	>50
T257-31	AG	1.16	1.21	4.87	1.58	4.55	>50

IC_{80} (µg/mL)
<0.001
.001-.01
.01-.100
.100-1.00
1.00-10.0
>10.0

Supplementary Figure 7

		Tri-NAb	Bi-ScFv	Bi-NAb	10E8	VRC01	PGT121
T266-60	AG	3.57	1.64	5.19	>50	5.78	0.620
T278-50	AG	>50	>50	>50	2.10	>50	>50
T280-5	AG	0.103	0.043	0.129	4.77	0.094	0.010
T33-7	AG	0.367	0.236	0.545	2.83	0.047	>50
3988.25	B	0.084	0.058	0.118	0.293	1.12	0.008
5768.04	B	0.318	0.108	0.245	5.26	0.956	0.897
6101.10	B	0.038	0.057	0.132	0.005	0.112	0.018
6535.3	B	0.031	0.029	0.122	1.28	5.77	0.011
7165.18	B	0.355	0.218	0.489	2.71	>50	0.074
45_01dG5	B	0.058	0.028	0.131	0.703	0.047	0.009
89.6.DG	B	0.052	0.410	0.444	1.48	2.03	0.077
AC10.29	B	0.549	0.475	1.21	0.512	2.76	0.118
ADA.DG	B	0.097	0.225	0.474	0.358	1.32	0.015
Bal.01	B	0.079	0.023	0.073	1.91	0.307	0.044
BaL.26	B	0.132	0.063	0.100	2.39	0.138	0.050
BG1168.01	B	1.68	5.61	5.88	1.48	3.57	>50
BL01.DG	B	22.8	>50	>50	1.57	>50	>50
BR07.DG	B	0.171	0.492	0.998	0.445	4.69	0.338
BX08.16	B	0.102	0.198	0.223	1.30	1.09	0.007
CAAN.A2	B	0.170	0.116	0.300	5.70	3.71	0.027
CNE10	B	0.094	0.065	0.207	0.169	1.59	0.027
CNE12	B	0.102	0.061	0.169	1.09	2.14	0.014
CNE14	B	0.071	0.035	0.109	0.649	0.813	0.007
CNE4	B	0.579	1.19	2.69	0.437	2.96	>50
CNE57	B	0.165	0.117	0.427	0.317	1.26	0.035
HO86.8	B	45.9	>50	>50	1.52	>50	>50
HT593.1	B	0.441	1.47	3.63	0.285	1.62	>50
HXB2.DG	B	0.028	0.019	0.166	0.015	0.093	>50
JRCSF.JB	B	0.452	0.126	0.747	1.89	0.925	0.219
JRFL.JB	B	0.156	0.062	0.223	0.768	0.087	0.071
MN.3	B	0.026	0.035	0.138	0.0010	0.063	>50
PVO.04	B	2.37	1.21	3.10	6.43	1.20	0.436
QH0515.01	B	1.91	1.08	2.99	5.54	2.85	>50
QH0692.42	B	1.59	2.41	4.40	2.35	3.98	9.70
REJO.67	B	0.254	0.105	0.415	1.18	0.180	>50
RHPA.7	B	0.246	0.102	0.300	5.10	0.112	0.046
SC422.8	B	0.934	0.474	1.10	1.15	0.321	0.362
SF162.LS	B	0.032	0.004	0.041	1.06	0.580	0.017
SS1196.01	B	0.014	0.051	0.144	1.25	0.679	0.011
THRO.18	B	1.13	6.69	12.5	0.587	9.91	>50
TRJO.58	B	1.02	0.777	2.25	4.18	0.231	>50
TRO.11	B	0.150	0.098	0.326	0.286	1.19	0.032
WITO.33	B	0.365	0.800	2.98	0.305	0.295	3.25
X2278.C2.B6	B	0.196	0.140	0.410	2.24	0.356	0.034
YU2.DG	B	0.181	0.111	0.500	5.46	0.188	0.178
BJOX002000.03.2	BC	0.273	0.129	0.337	1.56	>50	0.068
CH038.12	BC	0.094	0.109	0.241	1.41	1.04	0.020
CH070.1	BC	0.014	0.116	0.272	13.5	>50	0.015
CH117.4	BC	0.060	0.026	0.125	0.859	0.241	>50
CH119.10	BC	0.153	0.176	0.514	2.36	2.15	0.116
CH181.12	BC	0.253	0.134	0.334	2.79	1.22	0.039
CNE15	BC	0.062	0.235	0.709	2.97	0.375	>50
CNE19	BC	0.024	0.010	0.044	1.11	0.659	0.063
CNE20	BC	0.059	0.009	0.036	0.732	>50	0.008
CNE21	BC	0.129	0.081	0.236	3.25	0.903	0.018
CNE40	BC	0.048	0.855	1.29	0.009	4.55	1.85
CNE7	BC	0.206	0.146	0.558	0.603	0.605	0.096
286.36	C	0.054	0.023	0.085	5.00	0.715	0.009
288.38	C	0.022	0.106	0.176	3.08	3.46	0.035
0013095-2.11	C	0.025	0.639	2.57	0.077	0.287	>50
001428-2.42	C	0.144	0.062	0.217	6.28	0.035	0.076
0077_V1.C16	C	1.12	0.697	1.82	7.11	3.10	>50
00836-2.5	C	0.078	0.021	0.107	1.77	0.462	>50
0921.V2.C14	C	0.280	0.090	0.325	3.03	0.519	>50
16055-2.3	C	0.125	0.046	0.159	3.31	0.224	12.2
16845-2.22	C	0.389	11.2	22.6	0.172	12.3	>50
16936-2.21	C	0.030	0.033	0.111	1.31	0.435	0.013
25710-2.43	C	0.130	0.108	0.396	0.304	1.40	0.055
25711-2.4	C	0.160	0.081	0.242	1.69	1.44	0.041
25925-2.22	C	0.224	0.142	0.442	1.53	1.34	0.072
26191-2.48	C	0.543	0.331	0.953	4.90	0.679	0.393
3168.V4.C10	C	2.05	1.50	2.64	8.18	0.325	1.94

IC_{80} (µg/mL)
<0.001
.001-.01
.01-.100
.100-1.00
1.00-10.0
>10.0

Supplementary Figure 7

		Tri-NAb	Bi-ScFv	Bi-NAb	10E8	VRC01	PGT121
3637.V5.C3	C	9.98	37.1	>50	6.68	6.17	>50
3873.V1.C24	C	0.069	0.664	2.03	15.7	6.97	0.106
426c	C	1.20	0.967	2.48	1.60	4.41	>50
6322.V4.C1	C	12.0	>50	29.0	3.68	>50	>50
6471.V1.C16	C	>50	>50	>50	14.9	>50	>50
6631.V3.C10	C	8.27	6.28	9.28	3.36	>50	>50
6644.V2.C33	C	0.100	0.075	0.448	0.124	0.421	0.171
6785.V5.C14	C	0.383	0.138	0.390	2.42	0.686	0.072
6838.V1.C35	C	0.036	0.007	0.031	1.01	0.616	0.840
96ZM651.02	C	0.087	0.099	0.200	0.177	2.54	0.044
BR025.9	C	0.061	0.047	0.091	1.11	2.37	0.008
CAP210.E8	C	2.68	11.0	20.1	2.01	>50	>50
CAP244.D3	C	0.117	1.14	3.48	1.48	3.53	>50
CAP256.206.C9	C	0.141	0.138	0.308	2.97	2.72	0.045
CAP45.G3	C	0.483	0.398	0.963	3.41	>50	>50
Ce1176.A3	C	0.212	0.120	0.300	1.15	4.92	0.070
CE703010217.B6	C	0.053	0.022	0.087	0.679	0.584	0.011
CNE30	C	0.466	0.688	1.44	2.29	2.15	0.249
CNE31	C	2.93	2.23	7.20	3.57	2.10	2.68
CNE53	C	0.061	0.091	0.254	1.01	0.302	0.054
CNE58	C	2.46	3.09	5.78	1.09	0.582	>50
DU123.06	C	0.027	0.216	0.501	0.423	46.1	0.101
DU151.02	C	0.204	0.036	0.116	1.71	>50	0.021
DU156.12	C	0.045	0.030	0.117	0.120	0.188	0.023
DU172.17	C	0.197	0.244	0.480	0.238	>50	0.846
DU422.01	C	0.719	0.231	0.757	0.812	>50	0.365
MW965.26	C	0.024	0.044	0.127	0.007	0.128	0.051
SO18.18	C	0.008	0.013	0.061	4.48	0.085	0.006
TV1.29	C	3.39	0.897	1.80	0.719	>50	0.318
TZA125.17	C	3.14	7.29	22.1	1.19	>50	>50
TZBD.02	C	0.060	0.026	0.090	4.31	0.147	0.060
ZA012.29	C	0.165	0.055	0.129	4.12	0.602	0.021
ZM106.9	C	0.155	0.059	0.161	>50	0.513	0.018
ZM109.4	C	0.368	0.312	0.926	1.07	0.414	>50
ZM135.10a	C	0.093	3.95	7.79	0.408	6.10	9.25
ZM176.66	C	0.171	0.232	0.727	1.73	0.207	>50
ZM197.7	C	0.706	3.92	15.6	0.369	1.64	>50
ZM214.15	C	2.68	3.40	8.45	5.98	3.36	2.37
ZM215.8	C	0.083	0.066	0.281	0.230	0.937	0.057
ZM233.6	C	0.322	0.199	0.628	0.737	6.95	>50
ZM249.1	C	0.817	1.58	3.30	2.27	0.442	>50
ZM53.12	C	0.031	0.004	0.021	6.72	1.88	0.016
ZM55.28a	C	0.726	0.358	1.06	6.78	0.629	0.233
3326.V4.C3	CD	0.075	0.035	0.042	4.29	2.98	>50
3337.V2.C6	CD	0.178	0.119	0.241	4.87	0.211	>50
3817.v2.c59	CD	4.56	35.4	>50	1.43	>50	>50
191821.E6.1	D	3.01	1.15	3.89	5.89	1.34	>50
231965.c1	D	1.15	3.30	3.35	20.4	1.01	>50
247-23	D	2.06	>50	>50	1.29	12.3	>50
3016.v5.c45	D	0.118	0.086	0.186	2.17	0.252	>50
57128.vrc15	D	1.03	1.37	2.73	1.50	>50	>50
6405.v4.c34	D	0.357	0.255	0.645	1.80	4.24	0.080
A03349M1.vrc4a	D	0.462	0.184	0.433	0.663	13.9	0.155
A07412M1.vrc12	D	0.106	0.034	0.134	0.873	0.339	0.104
NKU3006.ec1	D	6.89	10.1	26.7	2.46	1.29	>50
UG021.16	D	0.103	0.929	1.18	0.362	1.59	>50
UG024.2	D	0.204	7.55	2.13	0.241	0.667	>50
P0402.c2.11	G	0.019	0.064	0.188	0.460	0.488	0.026
P1981.C5.3	G	0.072	0.018	0.054	0.124	0.691	0.013
X1193.c1	G	0.071	0.332	0.709	1.15	0.350	0.091
X1254.c3	G	0.040	0.159	0.535	15.7	0.132	0.069
X1632.S2.B10	G	0.251	0.322	0.582	1.76	0.526	>50
X2088.c9	G	0.126	0.046	0.097	>50	>50	0.009
X2131.C1.B5	G	0.076	0.080	0.197	0.175	1.41	0.044
SIVmac251.30.SG3	NA	>50	>50	>50	>50	>50	>50
SVA.MLV	NA	>50	>50	>50	>50	>50	>50

IC_{80} (µg/mL)
<0.001
.001-.01
.01-.100
.100-1.00
1.00-10.0
>10.0

Supplementary Figure 7

# Viruses	Tri-NAb	Bi-ScFv	Bi-NAb	1.00E+09	VRC01	PGT121
# Viruses	208	208	208	208	208	208
**Total Virus Neutralized**						
**IC80 <50 (µg/mL)**	204	196	194	203	182	112
**IC80 <10 (µg/mL)**	198	185	176	193	175	111
**IC80 <1.0 (µg/mL)**	155	144	122	61	106	99
**IC80 <0.1 (µg/mL)**	55	62	19	10	13	72
**IC80 <0.01 (µg/mL)**	1	4	0	5	0	11
**% Virus Neutralized**						
**IC80 <50 (µg/mL)**	98	94	93	98	88	54
**IC80 <10 (µg/mL)**	95	89	85	93	84	53
**IC80 <1.0 (µg/mL)**	75	69	59	29	51	48
**IC80 <0.1 (µg/mL)**	26	30	9	5	6	35
**IC80 <0.01(µg/mL)**	0	2	0	2	0	5
**Median IC80 (µg/mL)**	0.235	0.216	0.540	1.69	0.703	0.059
**Geometric Mean (µg/mL)**	0.298	0.299	0.749	1.34	0.793	0.081

IC_{80} (µg/mL)
<0.001
.001-.01
.01-.100
.100-1.00
1.00-10.0
>10.0

Supplementary Figure 7. Summary of IC_{80} (µg/ml) for trispecific and bispecific antibodies tested in 208 virus panel.

Supplementary Table 1. Summary of distances between the termini of PGT121 and VRC01 when bound to JR-FL SOSIP.664 trimer*

**Distance Between PGT121-VRC01 termini**				
**Inter-Protomer Orientation**				
VRC01	***PGT121***	***VRC01 C-terminus***	***PGT121 N-terminus***	***Distance (Å)***
VH-VL	VH-VL	VAL 106.V CB	GLN 1.H CB	60.663
VH-VL	VL-VH	VAL 106.V CB	ALA 6.L CB	48.913
VL-VH	VL-VH	VAL 111.U CB	ALA 6.L CB	37.171
VL-VH	VH-VL	VAL 111.U CB	GLN 1.H CB	66.444
**Orientation**				
PGT121	***VRC01***	***PGT121 C-terminus***	***VRC01 N-terminus***	***Distance (Å)***
VH-VL	VH-VL	VAL 106.L CB	GLN 1.U CB	53.470
VH-VL	VL-VH	VAL 106.L CB	VAL 3.V CB	63.459
VL-VH	VL-VH	SER 111.H CB	VAL 3.V CB	96.431
VL-VH	VH-VL	SER 111.H CB	GLN 1.U CB	77.125
**Intra-Protomer Orientation**				
VRC01	***PGT121***	***VRC01 C-terminus***	***PGT121 N-terminus***	***Distance (Å)***
VH-VL	VH-VL	VAL 106.V CB	GLN 1.H CB	106.920
VH-VL	VL-VH	VAL 106.V CB	PRO 7.L CB	100.662
VL-VH	VL-VH	VAL 111.U CB	ALA 6.L CB	73.361
VL-VH	VH-VL	VAL 111.U CB	GLN 1.H CB	78.762
**Orientation**				
PGT121	***VRC01***	***PGT121 C-terminus***	***VRC01 N-terminus***	***Distance (Å)***
VH-VL	VH-VL	VAL 106.L CB	GLN 1.U CB	96.925
VH-VL	VL-VH	VAL 106.L CB	VAL 3.V CB	94.519
VL-VH	VL-VH	SER 111.H CB	VAL 3.V CB	93.620
VL-VH	VH-VL	SER 111.H CB	GLN 1.U CB	94.350

* Inter-protomer and intra-protomer distances were inspected by Chimera, based on antibody-trimer complex structure (PDB: 5FYK). PGT122 serves as a surrogate for PGT121.
